# Extracting quantitative biological information from bright-field cell images using deep learning

**DOI:** 10.1063/5.0044782

**Published:** 2021-07-20

**Authors:** Saga Helgadottir, Benjamin Midtvedt, Jesús Pineda, Alan Sabirsh, Caroline B. Adiels, Stefano Romeo, Daniel Midtvedt, Giovanni Volpe

**Affiliations:** 1 Department of Physics, University of Gothenburg, Gothenburg, Sweden; 2 Advanced Drug Delivery, Pharmaceutical Sciences, R&D, AstraZeneca, Gothenburg, Sweden; 3 Department of Molecular and Clinical Medicine, Institute of Medicine, Sahlgrenska Academy, Wallenberg Laboratory, University of Gothenburg, Gothenburg, Sweden; 4 Department of Cardiology, Sahlgrenska University Hospital, Gothenburg, Sweden; 5 Clinical Nutrition Unit, Department of Medical and Surgical Sciences, University Magna Graecia, Catanzaro, Italy

## Abstract

Quantitative analysis of cell structures is essential for biomedical and pharmaceutical research. The standard imaging approach relies on fluorescence microscopy, where cell structures of interest are labeled by chemical staining techniques. However, these techniques are often invasive and sometimes even toxic to the cells, in addition to being time consuming, labor intensive, and expensive. Here, we introduce an alternative deep-learning–powered approach based on the analysis of bright-field images by a conditional generative adversarial neural network (cGAN). We show that this is a robust and fast-converging approach to generate virtually stained images from the bright-field images and, in subsequent downstream analyses, to quantify the properties of cell structures. Specifically, we train a cGAN to virtually stain lipid droplets, cytoplasm, and nuclei using bright-field images of human stem-cell–derived fat cells (adipocytes), which are of particular interest for nanomedicine and vaccine development. Subsequently, we use these virtually stained images to extract quantitative measures about these cell structures. Generating virtually stained fluorescence images is less invasive, less expensive, and more reproducible than standard chemical staining; furthermore, it frees up the fluorescence microscopy channels for other analytical probes, thus increasing the amount of information that can be extracted from each cell. To make this deep-learning–powered approach readily available for other users, we provide a Python software package, which can be easily personalized and optimized for specific virtual-staining and cell-profiling applications.

## I. INTRODUCTION

**FIG. 1.**
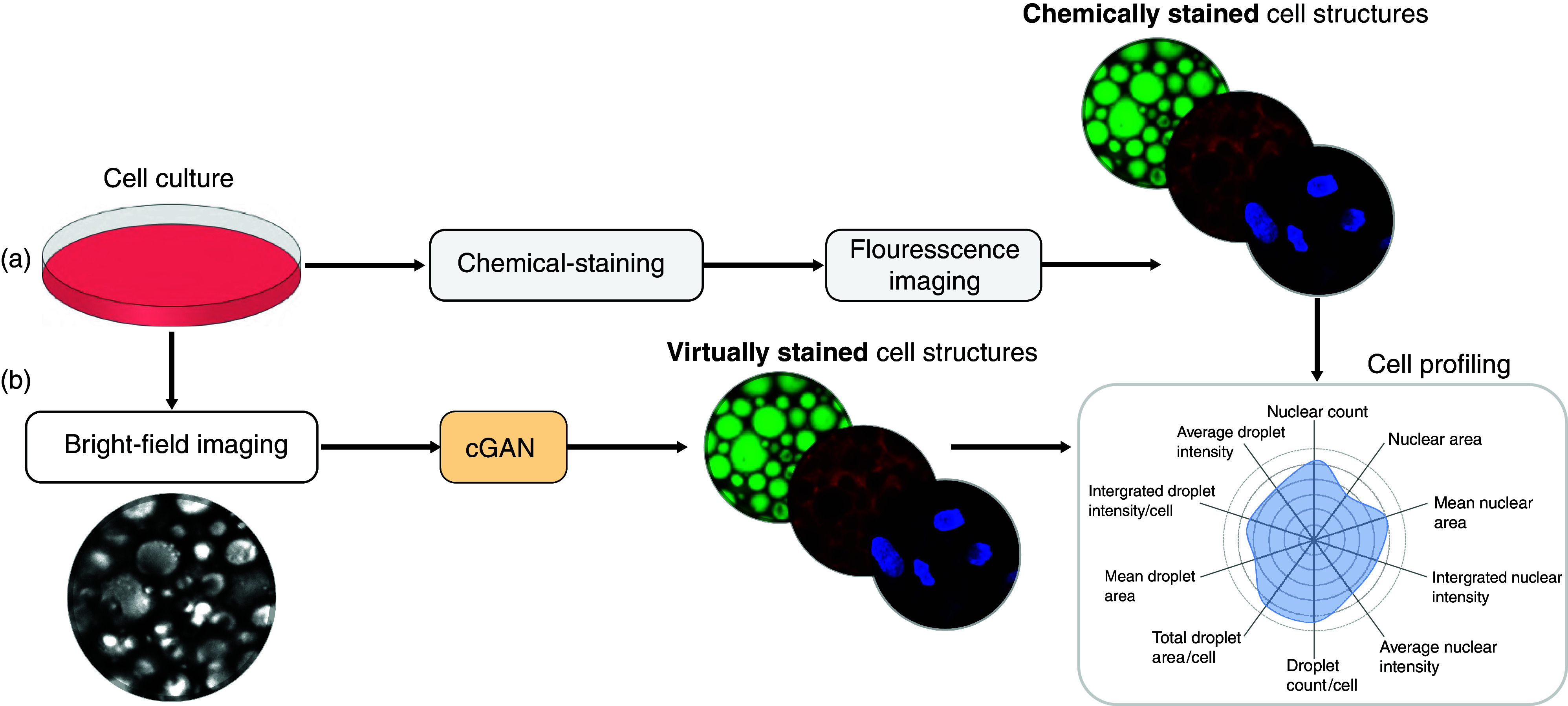
From cell cultures to quantitative biological information. (a) The standard workflow entails chemically staining the cell structures of interest, imaging them using fluorescence microscopy (in multiple light channels), and, finally, using these fluorescence images to retrieve quantitative biologically relevant measures about the cell structures of interest. (b) The deep-learning–powered approach we propose replaces the chemical-staining and fluorescence microscopy with a conditional generative adversarial neural network (cGAN) that uses bright-field images to generate virtual fluorescence-stained images.

Biomedical and pharmaceutical research often relies on the quantitative analysis of cell structures. For example, changes in the morphological properties of cell structures are used to monitor the physiological state of a cell culture,^1^ to identify abnormalities,^2^ and to determine the uptake and toxicity of drugs.^3^ The standard workflow is shown in Fig. 1(a): the cell structures of interest are chemically stained using fluorescence staining techniques; fluorescence images are acquired; and, finally, these images are analyzed to retrieve quantitative measures about the cell structures of interest. One key advantage is that multiple fluorescence images of the same cell culture can be acquired in parallel using the appropriate combination of chemical dyes and light filters, with the resulting images containing information about different cell structures.

However, fluorescence cell imaging has significant drawbacks. First, it requires a fluorescence microscope equipped with appropriate filters that match the spectral profiles of the dyes. Besides the complexity of the optical setup, usually only one dye is imaged at each specific wavelength, limiting the combination of dyes and cell structures that can be imaged in a single experiment. Second, the staining of the cell structures is typically achieved by adding chemical fluorescence dyes to a cell sample, which is an invasive (due to the required culture media exchange and dye uptake^4^) and sometimes even toxic process.^5^ Third, phototoxicity and photobleaching can also occur while acquiring the fluorescence images, which results in a tradeoff between data quality, time scales available for live-cell imaging (duration and speed), and cell health.^6^ Furthermore, for some dyes a cell-permeable form enters a cell and then reacts to form a stable and impermeable reaction product that is transferred to daughter cells; as a consequence, the dye intensity dilutes at every cell division and is eventually lost. Fourth, fluorescence staining techniques are often expensive, time consuming and labor intensive, as they may require long protocol optimizations (e.g., dye concentration, incubation, and washing times must be optimized for each cell type and dye). Also, care must be taken when choosing multiple dye partners to avoid spectral bleed-through.^7^ All these drawbacks aggravate, or hinder completely, the collection of reliable and long-term longitudinal data on the same population, such as when studying cell behavior or drug uptake over time. Therefore, there is an interest in extracting the same information using cheaper, noninvasive methods. In particular, it would be desirable to replace fluorescence images with bright-field images, which are much easier to acquire and do not require specialized sample preparation, eliminating concerns about the toxicity of the fluorescence dyes or damage related to the staining and imaging procedures. However, while bright-field images do provide some information about cellular organization, they lack the clear contrast of fluorescence images, which limits their use in subsequent downstream quantitative analyses.

Recently, the use of deep learning has been proposed as a way to create images of virtually stained cell structures, thus mitigating the inherent problems associated with the conventional chemical staining. These proposals come in the wake of the deep learning revolution,^8,9^ where convolutional neural networks have been widely used to analyze images, e.g., for microscopy,^10^ particle tracking,^11–14^ and the closely related problem of image-to-image cross-modality transformations.^15,16^ Virtually stained images have been created from images acquired with various imaging modalities. For example, virtual staining of cells, cellular components, and histopathology slides has been achieved using quantitative phase imaging,^17–19^ autofluorescence imaging,^20^ and holographic microscopy.^21^ Interestingly, the ability of reflectance microscopy to detect nanoscale structural changes beyond the diffraction limit has recently been exploited to generate virtually stained images for quantitative analysis of cell structures.^22^ Furthermore, more recent work suggests that the information required to reproduce different stainings is in fact available within bright-field images, even though the detail of these images are largely limited by diffraction.^6,15,23,24^

Here, we demonstrate a robust and fast-converging deep-learning–powered approach to transform bright-field images into virtually stained images, and, using these virtually stained images, we quantify the properties of cell structures. A high-level description of the proposed workflow is shown in Fig. 1(b). Specifically, we propose a conditional generative adversarial neural network (cGAN) that uses a stack of bright-field images of human stem-cell–derived adipocytes to generate virtual fluorescence-stained images of their lipid droplets, cytoplasm, and nuclei. We demonstrate that our network is robust and fast-converging in terms of quantitative biologically relevant measures extracted from the virtually stained images in a downstream cell-profiling analysis. Our method moves beyond seeking an optimal image-to-image transformation, exploiting the capabilities of adversarial generative models to extract relevant quantitative biological information. We apply this method to a dataset with three different magnifications (20×, 40×, and 60×), demonstrating that it also works in conditions where some of the biological features are not clearly visible in the bright-field images. In order to make this deep-learning–powered approach readily available for other users, we provide a Python software package, which can be easily personalized and optimized for specific virtual-staining and cell-profiling applications.^25^

### A. Virtually stained fluorescence images from bright-field images

We employ a cGAN^26^ to generate virtually stained fluorescence images of lipid droplets, cytoplasm, and nuclei from a z-axis stack of confocal bright-field images (each image is 280 *μ*m × 230 *μ*m, 2560 × 2160 pixels). We describe in detail the data in Appendix A and the deep learning architecture in Appendix B. Briefly, our cGAN consists of two networks:^26^ a generator, which receives as input a stack of bright-field images and generates virtually stained fluorescence images, and a discriminator, which determines whether images are authentic (i.e., fluorescently stained samples) or created by the generator. These two neural networks are trained simultaneously. The generator progressively becomes better at generating virtually stained images that can fool the discriminator. In turn, the discriminator becomes better at discriminating chemically stained images from generated images. More details about the training procedure are in Appendix C.

**FIG. 2.**
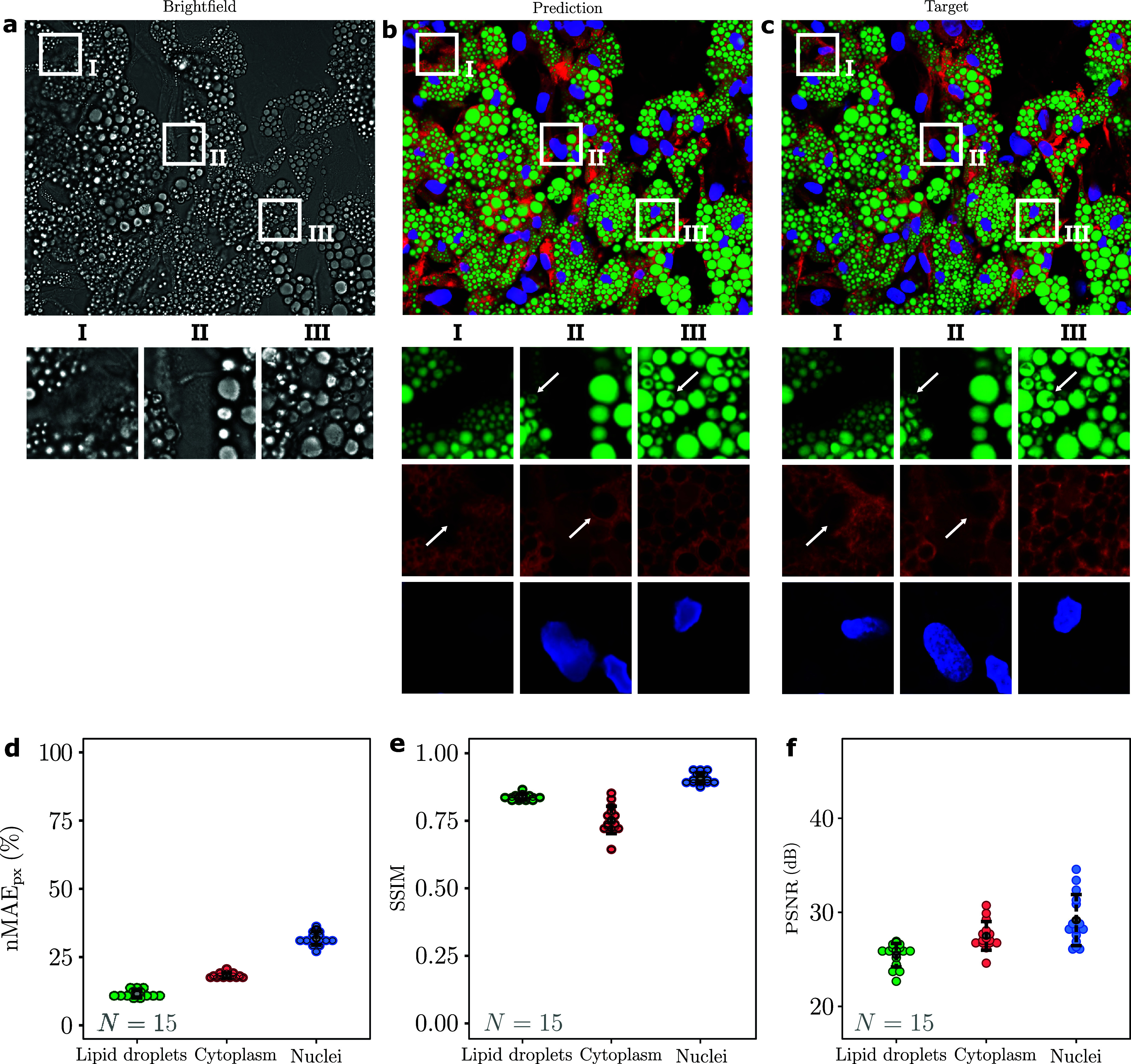
Visualization and quantitative evaluation of virtually stained fluorescence images (60× magnification). (a) Bright-field image and corresponding merged (b) virtually stained and (c) chemically stained fluorescence images for lipid droplets (green), cytoplasm (red) and nuclei (blue), and corresponding enlarged crops (I, II, and III). The lipid droplets are clearly visible in the bright-field image (a) thanks to their high refractive index so that the cGAN manages to generate accurate virtual stainings [e.g., green crops in (b)] corresponding to the chemically stained images [e.g., green crops in (c)], even reproducing some details of the internal structure of the lipid droplets (darker areas in the droplets indicated by the arrows). Also, the virtual staining of the cytoplasm [e.g., red crops in (b)] closely reproduces the corresponding chemical staining [e.g., red crops in (c)]; this is particularly evident in the contrast between various cytoplasmic structures (indicated by the arrows). The virtually stained nuclei [e.g., blue crops in (b)] deviate more prominently from the chemically stained ones [e.g., blue crops in (c)], especially in the details of both their shape and texture, which can be explained by the fact that the nuclei are not clearly visible in the bright-field image so that the cGAN seems to use the surrounding cell structures to infer the presence and properties of the nuclei shape. To quantify the quality of the virtually stained images, (d) the pixel-value normalized mean absolute error for the pixel values (nMAE_px_), (e) the structural similarity index measure (SSIM), and (f) the peak signal-to-noise ratio (PSNR) of all validation images (*N *=* *15) for lipid droplets (green), cytoplasm (red), and nuclei (blue) are calculated (each colored circle represents one validation image, while the black circle represents the mean over all images).

Figures 2(a)–2(c) present a representative validation result of virtual staining for 60× magnification (results for all validation data and all magnifications are available on our Github repository^25^). Figure 2(a) shows the first of the seven bright-field slices used as input for the cGAN. The corresponding virtually stained and chemically stained fluorescence images are shown in Figs. 2(b) and 2(c), respectively. Comparing the bright-field input [Fig. 2(a)] with the fluorescence targets [Fig. 2(c)], we can see that, while the bright-field image contains information about the cellular structures, such information is less readily accessible than in the fluorescence images. Furthermore, different cell structures have distinct prominence in the bright-field image, with the lipid droplets being more clearly visible than the cytoplasm, and in turn the cytoplasm more evident than the nuclei. Nevertheless, despite the limited information in the bright-field images, the cGAN manages to predict fluorescence images [Fig. 2(b)] that are qualitatively similar to the chemically stained images [Fig. 2(c)].

The lipid droplets are virtually stained with great detail, as can be appreciated by comparing the enlarged crops of the virtual staining [green crops in Fig. 2(b)] with those of the chemical staining [green crops in Fig. 2(c)]. Since the lipid droplets consist primarily of lipids at high concentration, they have a higher refractive index than most other intracellular objects,^27^ which makes them visible in the bright-field images and allows predicting high-quality structural features. Interestingly, even some details about the internal structure of the lipid droplets can be seen in the virtual staining [e.g., the darker areas inside the droplets indicated by the arrows in the green crops II and III in Figs. 2(b) and 2(c)]. These structures are probably due to proteins embedded in the surface or core of the droplets that affect the appearance of the chemically stained cells:^28^ Since most of the space inside adipocytes is occupied by lipid droplets, when these cells need to increase their metabolic activity (e.g., during protein synthesis), they rearrange their contents, creating textural imprints on the surfaces of the lipid droplets resulting in golf-ball–like textures.

Similar to the lipid droplets, the cytoplasm virtually stained images exhibit a high-quality reconstruction, as can be seen by comparing the corresponding enlarged virtually stained images [red crops in Fig. 2(b)] with the corresponding enlarged chemically stained images [red crops in Fig. 2(c)]. Some of the fine structures appear to be slightly different, namely, the contrast between various cytoplasmic structures [see, e.g., those indicated by the arrows in the red crops I and II in Figs. 2(b) and 2(c)]. However, since the cytoplasm dye (CellTracker Deep Red) reacts with amine groups present in intracellular proteins dispersed in the cytoplasm, this probably leads to uneven staining patterns in the chemically stained images, which are intrinsically random and not reproducible by the virtual-staining procedure.

The nuclei are more challenging to virtually stain because they have a similar refractive index to the surrounding cytoplasm,^29^ so there is limited information about them in the bright-field image. Nevertheless, the cGAN manages to identify them, as can be seen by comparing the enlarged crops of the virtual staining [blue crops in Fig. 2(b)] with the corresponding chemically stained nuclei [blue crops in Fig. 2(c)], although without resolving the details of their internal structure. The cGAN seems to extract information about the nuclei shape primarily based on the surrounding cell structures, making it difficult to predict nuclei that are not surrounded by lipid droplets. Despite this, the cGAN manages to identify the nuclei reliably. Considering that the cell is typically at its thickest around the position of the nucleus, complementing the bright-field images with phase-contrast images may give additional information that helps to increase the robustness of the virtual nuclei staining.

To quantify the quality of the virtually stained images, we calculated the pixel-wise normalized mean absolute error [nMAE_px_, Fig. 2(d)], the structural similarity index measure [SSIM, Fig. 2(e)], and the peak signal-to-noise ratio [PSNR, Fig. 2(f)] between the virtually stained images and chemically stained fluorescence labels for the 60× validation set (for further information regarding these metrics and why they were chosen, please refer to Appendix E). Results for 40× and 20× magnifications are available in the supplementary material (Figs. S1 and S2). This analysis reveals that the virtually stained images of lipid droplets exhibit the best performance in terms of the nMAE_px_ with an average nMAE_px_ equal to 0.12 ± 0.013 [green symbols in Fig. 2(d)]. The average nMAE_px_ for the virtually stained cytoplasm [red symbols in Fig. 2(d)] and nuclei [blue symbols in Fig. 2(d)] images are 0.18 ± 0.098 and 0.32 ± 0.025, respectively. The nMAE_px_ is an estimate of the pixel intensity errors, which is a relevant metric since most of the subsequent cytometric analysis is intensity dependent, as further explained in Sec. II B. However, low nMAE_px_ values do not necessarily imply high-quality predictions. Accordingly, we chose to evaluate further the results concerning the images' structural information using the SSIM. We obtained an average SSIM of 0.84 ± 0.011, 0.75 ± 0.051, and 0.91 ± 0.020 for the virtually stained images of lipid droplets [green symbols in Fig. 2(e)], cytoplasm [red symbols in Fig. 2(e)], and nuclei [blue symbols in Fig. 2(e)], respectively, demonstrating an accurate reproduction of the structural information with SSIM > 0.75. It is important to highlight that the cGAN preserves the structural features in the virtually stained images of nuclei better than intensity-based features. Finally, the cGAN achieves a high-quality reconstruction for the three cell substructures with an average PSNR of 26 ± 1.2, 27 ± 1.6, and 29 ± 2.8 dB for the virtually stained images of lipid droplets [green symbols in Fig. 2(f)], cytoplasm [red symbols in Fig. 2(f)], and nuclei [blue symbols in Fig. 2(f)], respectively.

The results for the 40× (supplementary material Fig. S1) and 20× magnification (supplementary material Fig. S2) show a similar trend to the 60× results above, but with increasing values for the nMAE_px_ and PSNR, and a more widely distributed PSNRs for the cytoplasm. The SSIM values for the lipid droplets decrease (from 0.84 to 0.76) but increase for cytoplasm (from 0.75 to 0.88), while being relatively stable for nuclei, with lowering magnifications. It is likely that the loss in detail for lower magnifications affects the smaller lipid droplets more than the larger cytoplasm structures, for which the lower magnifications, with a corresponding increase in depth of field, can even be beneficial.

### B. Extracting biologically relevant features from virtually stained images

The stained images are used to extract quantitative biological information about the cell structures. In fact, measurement of accurate and relevant quantitative cell structure data is of key importance for biomedical and pharmaceutical research as well as for clinical therapeutic decisions. For example, quantitative information about the cellular lipid droplet content is critical to study metabolic diseases where the fat storage in adipocytes plays a pivotal role and to dissect the mechanisms leading to organ injury due to lipid deposition in ectopic tissue.^31^ These kinds of experiments most often rely on the comparison of different samples, making the correlation of results from chemically and virtually stained images of higher importance than absolute values.

**FIG. 3.**
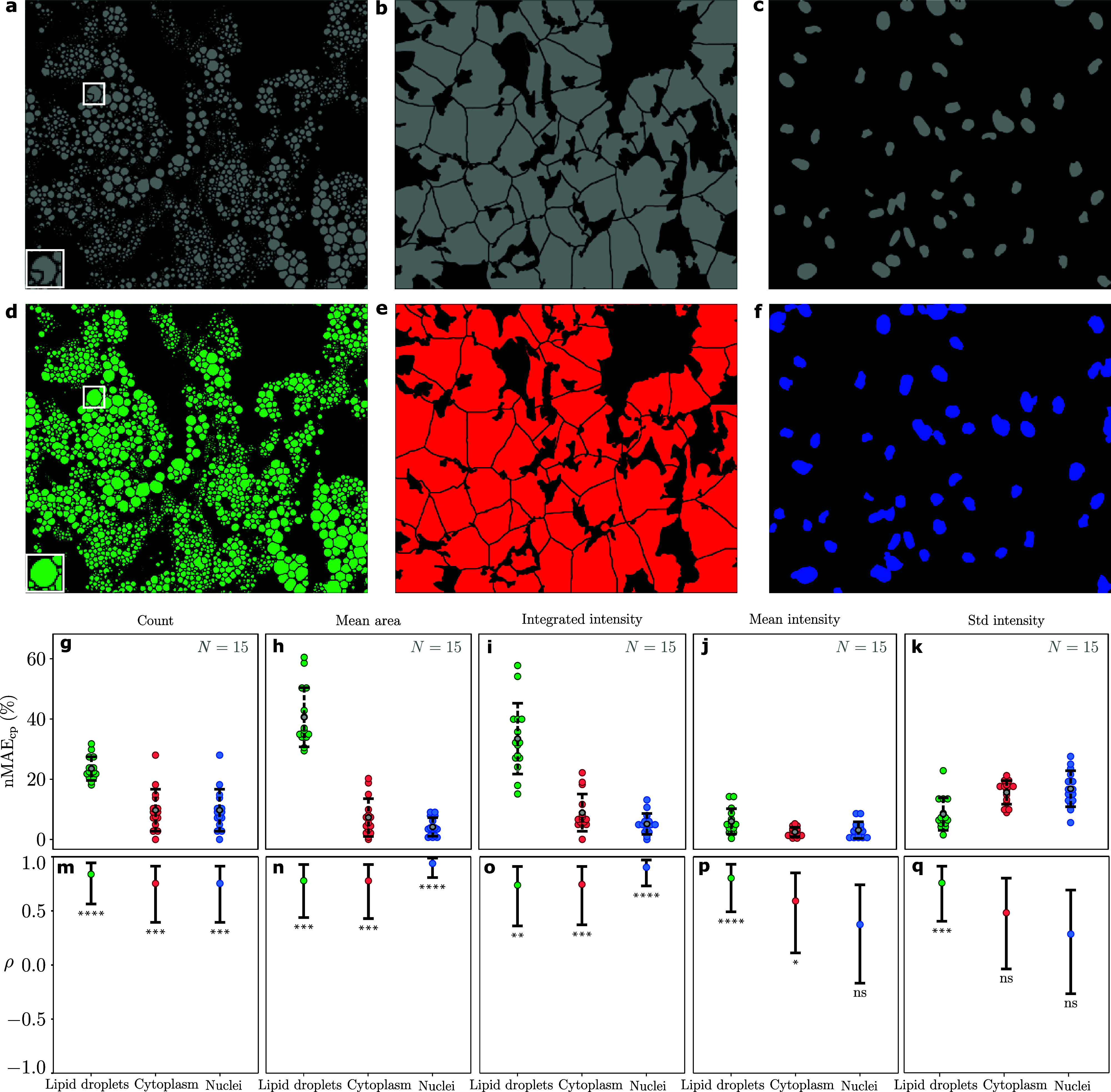
Quantitative evaluation of the biologically relevant features extracted from chemically stained and virtually stained fluorescence images (60× magnification). Segmentation obtained using CellProfiler (https://cellprofiler.org, version 4.07^30^) of (a)–(c) chemically stained target images and (d)–(f) virtually stained generated images for lipid droplets, cytoplasm, and nuclei. (g)–(k) Normalized mean absolute error between the features identified by CellProfiler in the virtually stained images compared to the chemically stained images (nMAE_cp_): (g) the difference in the number of cell structures counted in the images, (h) their mean area, (i) their combined integrated intensity over the image, (j) the mean intensity of cell structures in the image, and (k) the standard deviation of the mean intensity. (m)–(q) Pearson correlation coefficient (*ρ*) and the 95% confidence interval between the metrics obtained with the chemically stained and virtually stained images. Statistical significance levels obtained by two-tailed unpaired Student *t* test: ns, no statistical significance (*p *>* *0.05); ^*^p ≤ 0.05; ^**^p ≤ 0.01; ^***^p ≤ 0.001; *p* ≤ 0.0001.

Here, we have used the open-source software CellProfiler (version 4.07^30^) to identify and segment the lipid droplets, cytoplasm, and nuclei in both the chemically stained and virtually stained fluorescence images (the analysis pipeline is available on our Github repository^25^). For each cell structure, we employ a feature-extraction pipeline that calculates the number of cell structures in each image, their mean area in pixels, their integrated intensity, their mean intensity, and the standard deviation of their mean intensity. The results of this quantitative analysis are shown in Fig. 3 for the same representative set of validation images used in Fig. 2 (the results for all validation data are available on our Github repository^25^). The values of the aggregated results for the whole validation dataset are presented in Table I.

**TABLE I.** Comparison of features extracted from chemically stained and virtually stained images for the whole validation dataset (60× magnification). Average and standard deviation of various metrics (pixel value, count, mean area, integrated intensity, mean intensity, and standard deviation of the mean intensity of lipid droplets, cytoplasmic regions, and nuclei) calculated over the 15 sets of target chemically stained images and of the predicted virtually stained images of the validation dataset. We also report the absolute and normalized values of the mean absolute error (MAE and nMAE) as well as the correlation between the metrics calculated on the target and predicted images. Note that the pixel values are in the original image range [0, 65 535], while the intensity measurements are extracted with CellProfiler using images with intensities rescaled from 0 to 1. The features that are most biologically relevant for each cell structure are highlighted (in green, red, and blue for lipid droplets, cytoplasm, and nuclei, respectively).

Metrics	Target	Prediction	MAE	nMAE (%)	*ρ*
Lipid droplets					
Pixel-value	1300 ± 180	1300 ± 150	150 ± 36	12 ± 1.3	0.98
Count	6600 ± 580	5000 ± 260	1600 ± 390	24 ± 3.9	0.84
Mean area	400 ± 41	550 ± 36	160 ± 26	41 ± 9.8	0.75
Integrated intensity	13 ± 1.7	17 ± 1.7	4.2 ± 1.2	34 ± 12	0.74
Mean intensity	0.025 ± 0.0027	0.023 ± 0.0018	0.0016 ± 0.0013	6.0 ± 4.2	0.80
Std intensity	0.0035 ± 0.00034	0.0037 ± 0.00029	0.00027 ± 0.00016	8.1 ± 5.4	0.76
Cytoplasm					
Pixel-value	320 ± 9.9	330 ± 7.7	59 ± 2.8	18 ± 1.0	0.77
Count	34 ± 5.8	33 ± 4.0	3.1 ± 2.3	9.7 ± 7.0	0.75
Mean area	79000 ± 10000	82000 ± 11000	5800 ± 5000	7.3 ± 6.3	0.77
Integrated intensity	430 ± 53	460 ± 61	41 ± 28	9.3 ± 6.1	0.74
Mean intensity	0.0055 ± 0.00015	0.0056 ± 0.00013	0.00013 ± 0.000085	2.5 ± 1.6	0.59
Std intensity	0.0016 ± 0.000083	0.0014 ± 0.000034	0.00025 ± 0.000073	15 ± 3.9	0.48
Nuclei					
Pixel-value	290 ± 18	300 ± 16	92 ± 7.6	32 ± 2.5	0.82
Count	34 ± 5.8	33 ± 4.0	3.1 ± 2.1	9.7 ± 7.0	0.75
Mean area	7400 ± 1100	7100 ± 1100	320 ± 240	4.2 ± 3.1	0.93
Integrated intensity	170 ± 24	170 ± 26	9.4 ± 5.6	5.6 ± 3.4	0.90
Mean intensity	0.022 ± 0.00081	0.022 ± 0.00084	0.00068 ± 0.00060	3.1 ± 2.8	0.37
Std intensity	0.0065 ± 0.00042	0.0054 ± 0.00026	0.0011 ± 0.00043	16 ± 6.0	0.28

The first step of the feature-extraction pipeline is to segment the relevant cell structures. Starting from the fluorescence images, the feature-extraction pipeline identifies relevant cellular structures based on threshold values for intensity, size, and shape. Figures 3(a)–3(c) show the segmentations obtained from the chemically stained images, and Figs. 3(d)–3(f) the corresponding segmentations obtained from the virtually stained images.

In the feature-extraction pipeline, the nuclei are identified first [Figs. 3(c) and 3(f)]. Since the lipid droplets in the adipocytes may occlude the nuclei and physically change their size and shape, a wide range of possible nuclear diameters and shapes is selected to ensure a successful segmentation. Furthermore, since the intensity of the nuclei varies, an adaptive thresholding strategy is chosen (i.e., for each pixel, the threshold is calculated based on the surrounding pixels within a given neighborhood). As a last step, nuclei that are clumped together are distinguished by their shape. Identifying the nuclei is critically important because the number of nuclei is often used for the quantification of different biological phenomena, for example, the average amount of lipids per cell in the context of diabetes research.

In the second part of the feature-extraction pipeline, the cytoplasm is segmented to determine the cell boundaries, starting from the locations of the previously identified nuclei [Figs. 3(b) and 3(e)]. An adaptive thresholding strategy is again used, with a larger adaptive window (the neighborhood considered for the calculation of the threshold) compared to that used for the nuclei segmentation. Identifying the cytoplasm structure is important because it gives information about the cell size (measured area) and morphology (e.g., presence of protrusions or blebbing features), which are in turn related to the physiological state of the cell.^32^

In the final part of the feature-extraction pipeline, the lipid droplets are segmented independently from the nuclei and cytoplasm [Figs. 3(a) and 3(d)]. This segmentation is done in two steps to target separately the smaller and larger lipid droplets. For each of the two steps, a range of expected diameters and intensities are selected for the image thresholding. Since lipid droplets in each of the size distributions have similar peak intensities, a global thresholding strategy is used for their identification. Lipid droplets that are clumped together are distinguished by their intensity rather than their shape, which is consistently round for all the lipid droplets.

The segmented images are then used to count and characterize the cell structures. Figures 3(g)–3(k) show the distribution and normalized mean absolute error (nMAE_cp_, calculated for each feature in each image by normalizing the feature MAE by the true target value) of the biologically relevant features identified by CellProfiler between the virtually stained and chemically stained for the whole 60× validation dataset in terms of the cell structure count in the image, their mean area, their combined integrated intensity over the image, the mean intensity of cell structures in the image, and the standard deviation of the mean intensity. The amount of acceptable variance between the virtually and chemically stained images is dependent on the application at hand. However, Figs. 3(m)–3(q) show that there is a high correlation (Pearson correlation coefficient *ρ*) between all metrics obtained with the chemically stained and virtually stained images. This indicates that any deviation between these metrics is systematic and consistent, which is highly relevant for biological experiments, where the focus is not on absolute values but rather on the comparison of different samples. The values of the aggregated results for the features extracted using CellProfiler for the whole validation dataset are presented in Table I.

The feature extraction from the virtually stained images shows the most consistent performance for the lipid droplets. This is very useful for potential applications because lipid droplets are often used, e.g., to measure the effect of drugs for metabolic diseases. In this context, the amount of fat in cells is often quantified by normalizing the number of lipid droplets, their mean area, or integrated intensity to the number of cells in the image. In addition, the size, texture, location, and number of droplets can also be used to create phenotypic profiles that can reveal the effects of drugs on adipocyte physiology. A systematically lower number (*ρ* = 0.84) of larger lipid droplets (*ρ* = 0.75) is identified in the segmented virtually stained images [Fig. 3(d)] compared to the segmented chemically stained images [Fig. 3(a)]. This can be partly explained by the fact that chemically stained fluorescence images of the lipid droplets have some intensity variations [see, e.g., those indicated by the arrows in the green crops II and III in Fig. 2(c)], which may result in the “over-segmentation” of a single lipid droplet into multiple parts [see, e.g., the inset in Fig. 3(a)]. Even though these intensity variations are reproduced in the virtually stained images [see, e.g., those indicated by the arrows in the green crops II and III in Fig. 2(b)], they do not translate into an over-segmentation of the image by CellProfiler, leading to identification of fewer but larger lipid droplets [see, e.g., the inset in Fig. 3(d)]. Therefore, the lipid droplet count is lower, their area larger, and their integrate intensity is higher when analyzing the virtually stained images compared to when analyzing the chemically stained ones [green symbols in Figs. 3(g)–3(i) and 3(m)–3(o), and Table I]. Nevertheless, the average and standard deviation of their mean intensity are more closely estimated (nMAE_cp_ < 10% and *ρ* > 0.75), probably thanks to the fact that these are intensive quantities [green symbols in Figs. 3(j)–3(k) and 3(p)–3(q), and Table I].

The main information extracted from the cytoplasm staining is related to the cell boundaries and morphology. In this respect, the cell count and mean area are the most important metrics, which are reproduced very well by the analysis of the virtually stained images [nMAE_cp_ < 10% and *ρ* > 0.75, red symbols in Figs. 3(g)–3(h) and 3(m)–3(n), and Table I]. The other metrics are related to the intensity of the cytoplasm, which can be inconsistent even in the chemically stained images because the cytoplasmic dye (CellTracker Deep Red) reacts with amine groups present in intracellular proteins dispersed in the cytoplasm, producing an uneven texture. This explains why the cGAN cannot predict the exact spatial distribution and amount of the chemical dye from which the chemically stained images are obtained. On the other hand, the metrics about the integrated intensity, mean intensity, and standard deviation of the mean intensity are reproduced accurately, in terms of error values, from the virtually stained images, but with a lower correlation for the mean intensity and standard deviation of the mean intensity, meaning that the predictions are less consistent (red symbols in Figs. 3(i)–3(k) and 3(o)–3(q), and Table I).

The nuclei are used to identify the individual cells, for which both the number and morphological properties of the nuclei are needed. In this respect, the most important measures are the nuclei count and mean area, which are determined accurately, and consistently, using the virtually stained images [nMAE_cp_ < 10% and *ρ* = 0.75, and nMAE_cp_ < 5% and *ρ* = 0.93, respectively, blue symbols in Figs. 3(g)–3(h) and 3(m)–3(n), and Table I], as well as the integrated intensity [nMAE_cp_ < 6% and *ρ* = 0.90, blue symbols in Figs. 3(i) and 3(o), and Table I]. The other metrics (mean intensity and standard deviation of the intensity) are less consistently comparable to the chemically stained fluorescence images [Figs. 3(j)–3(m) and Table I]. The cGAN does not manage entirely to capture the dynamic content of the nuclei, possibly because of the non-static chromatin conformations present in living cells, resulting in different levels of dye accessibility. With this information not being visible in the bright-field images, it is not surprising that the virtual staining does not include textural details. Nevertheless, this is not generally a problem because in most studies the cell nuclei morphology or chromatin conformation is not the aim of the study, rather, the nuclei are often used to count cells for the purposes of normalization. The virtual staining does offer sensitive cell number determination and, as such, enables cell–cell comparison of other measured parameters. Considering the known phototoxicity of Hoechst 33342 in time-lapse imaging series of living cells,^33^ and if the nuclear stain is solely intended to enable nuclear counts and cell segmentation, the cGAN manages to perform this task and may be preferred.

Compared to the 60× magnification, the results for the 40× magnification (supplementary material Fig. S3) show a substantial decrease in the error for the number of lipid droplets (from about 24% to 12%), their mean area (41% to 18%) and integrated intensity (34% to 19%), and increase in correlation of these measures (0.83 to 0.93, 0.77 to 0.97, and 0.74 to 0.89, respectively). The same goes for the correlation of the number of nuclei and cytoplasm structures (0.75 to 0.91) and cytoplasm mean area (0.77 to 0.84), while the error remains similar. It seems like the decrease in detail in the images is favorable for the downstream analysis, as there might be fewer variations between the virtually and chemically stained images. For the 20× magnification (supplementary material Fig. S4), the error is still lower with a higher correlation for the number of lipid droplets (from about 24% to 9%) and their mean area (41% to 13%). The results for most of the other measures are worse compared to both 40× and 60× magnifications.

### C. Robustness and fast convergence of cGAN compared to U-Net

**FIG. 4.**
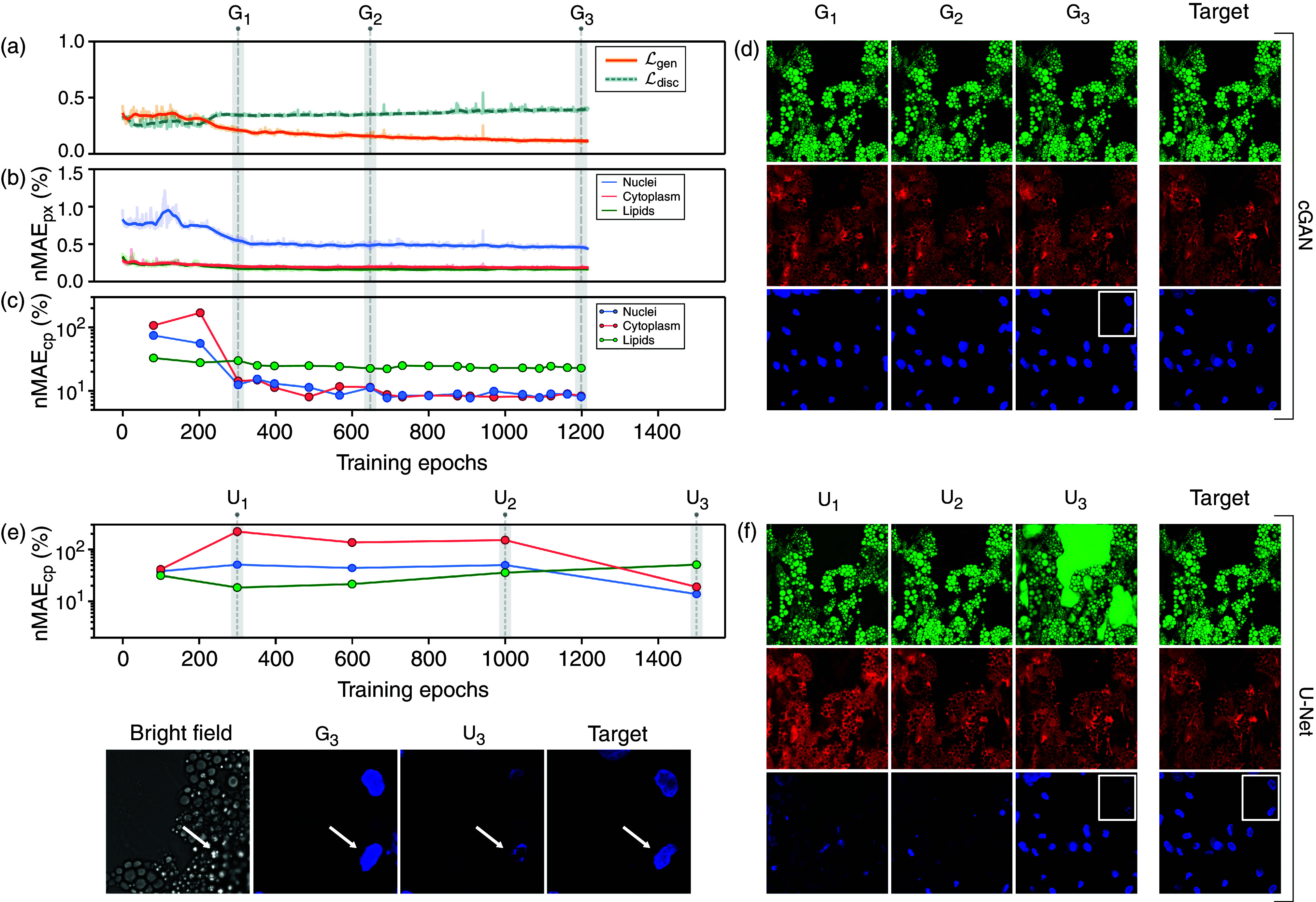
The cGAN is robust and fast-converging in terms of biologically relevant features (60× magnification). (a) Loss function of generator $\ell_{\text{gen}}$ and discriminator $\ell_{\text{disc}}$ of the cGAN, (b) validation pixel-wise nMAE loss (nMAE_px_), and (c) nMAE for the biological features extracted with CellProfiler (nMAE_cp_) for lipids, cytoplasm, and nuclei as a function of the training epoch. (d) The virtually stained fluorescence images of lipid droplets (green), cytoplasm (red), and nuclei (blue) generated by the cGAN at different number of epochs [corresponding to G_1_, G_2_, and G_3_ in (a)–(c)], demonstrating that the cGAN converges rapidly and provides consistently robust results throughout the training process. (e) nMAE for the biological features extracted with CellProfiler (nMAE_cp_) for the U-Net for lipid droplets (green), cytoplasm (red), and nuclei (blue). (f) Virtually stained fluorescence images for lipid droplets (green), cytoplasm (red), and nuclei (blue) generated by the U-Net at different number of epochs [corresponding to U_1_, U_2_, and U_3_ in (e)]. (g) The cGAN (*G*_3_) manages to identify the nuclei faster and more consistently compared to the U-Net (U_3_), even though there is little information about the nuclei in the bright-field images (indicated by the bright arrows).

Analyzing how the network evolves over the course of training for the 60× magnification, Fig. 4(a) reveals that the generator loss is continuously decreasing over the entire training time, with the most gain seen over the first 300 epochs. Similarly, Fig. 4(b) shows that the nMAE_px_ for the pixel values of the three channels improves significantly, especially in the first 300 epochs. However, Fig. 4(c) might be the most important, showing that the nMAE_cp_ for the biological features extracted with CellProfiler decreases only very marginally after 300 epochs, indicating that the network is able to learn the biologically relevant features very quickly. This is underscored by Fig. 4(d), which demonstrates that the network is very quickly able to generate images that are extremely similar in structure to the target chemically stained images. Since all validation metrics continue to decrease during the course of training, the model is unlikely to be overtrained.

The cGAN architecture and, in particular, the discriminator are crucial to the quality of the virtually stained images. Even though comparing different deep-learning architectures is tricky because hyperparameter optimization can lead to significant differences in end performance, it is a crucial control. Training the generator by simply removing the discriminator is not a fair comparison, because the generator is designed with a discriminator in mind. Instead, we demonstrate the advantages of a GAN-based architecture over a more traditional U-Net architecture by adapting the U-Net proposed in Ref. 6, which has been shown to be able to virtually stain several intracellular structures in bright-field images. In order to use this U-Net with our dataset, only two adaptations were required: to change the shape of the data expected by the model, and not to z-score normalize the targets in order to allow quantitative comparisons between the architectures. One such U-Net was trained for each of the three output features, similarly to the original paper,^6^ using the same training parameters as the cGAN. The resulting U-Net training process [Fig. 4(e)] is very different than that of the cGAN [Figs. 4(a)–4(c)]: it takes significantly longer for the U-Net to start converging in the terms of extracted biological features, and the lipid droplet channel at some point around 300 epochs starts diverging, which may be caused by overtraining. In Fig. 4(f), one can see the same stark contrast for the images virtually stained with the U-Net. As Fig. 4(g) exemplifies, even though there is little information about the nuclei in the bright-field images, the cGAN rapidly learns to accurately reproduce the fluorescently stained images, especially when compared to the U-Net. In fact, this is exactly what is expected, given that the cGAN is trained to match the output distribution, while the U-Net is trained to draw from a distribution that minimizes the mean square error. In other words, if sufficient information is lacking, the cGAN is trained to predict physically plausible images, while the U-Net is trained to predict a point in the output space somewhere in between all possible scenarios, resulting in physically impossible images. Figure 4(g) demonstrates this exact scenario, where a nucleus in a difficult region in the bright-field image (indicated by the arrow) is correctly found by the cGAN, while the U-Net predicts something in between a nucleus and a non-nucleus.

The training evolves similarly for the cGAN for 40× (supplementary material Fig. S5) and 20× (supplementary material Fig. S6) magnifications. The generator loss continuously decreases, with the most gain over only the first 100 epochs, converging even faster than for 60× magnification. After that, the nMAE for the pixel values and biological features extracted with CellProfiler only marginally decrease. This indicates that the cGAN quickly learns to generate images that are very similar in structure to the target chemically stained images and provides consistently robust results throughout the training process, independent of magnification.

We have developed a deep-learning–powered method for quantitative analysis of intracellular structures in terms of their size and morphology. The method is a robust and fast-converging approach based on virtually stained images of cells derived from bright-field images and subsequent downstream analysis to quantify the properties of the virtually stained cell structures.

We have demonstrated the accuracy and reliability of our method by virtually staining and quantifying the lipid droplets, cytoplasm, and cell nuclei from bright-field images of stem-cell derived adipocytes for three magnifications (60×, 40×, and 20×). While the lipid droplets are easily visible in the bright-field images, direct quantification of their size and content using conventional analysis techniques is challenging, and fluorescent staining techniques are typically used. The cytoplasm and cell nuclei are almost indistinguishable based on their optical contrast, but in this case the spatial distribution of the lipid droplets guides the network to correctly localize these structures.

Compared to standard approaches based on fluorescent staining, our approach is less labor intensive, and its results do not depend on careful optimization of the staining procedure or on the illumination parameters. Therefore, the results are more robust and can potentially be compared across experiments and even across labs. We note also that the proposed approach is not limited to the structures quantified in this work but can be applied to virtually stain and quantify any intracellular object with unique optical characteristics. Furthermore, virtual staining does not exclude fluorescent imaging, so additional information can also be obtained from the liberated fluorescence channels, such as particle uptake or protein expression, both of which are important, e.g., for studying and visualizing subcutaneous dosing of nanomedicines and vaccines.

To make this method readily available for future applications, we provide a Python open-source software package, which can be personalized and optimized for the needs of specific users and applications.^25^

## SUPPLEMENTARY MATERIAL

See the supplementary material for additional figures and analysis.

## Figures and Tables

**FIG. 5.**
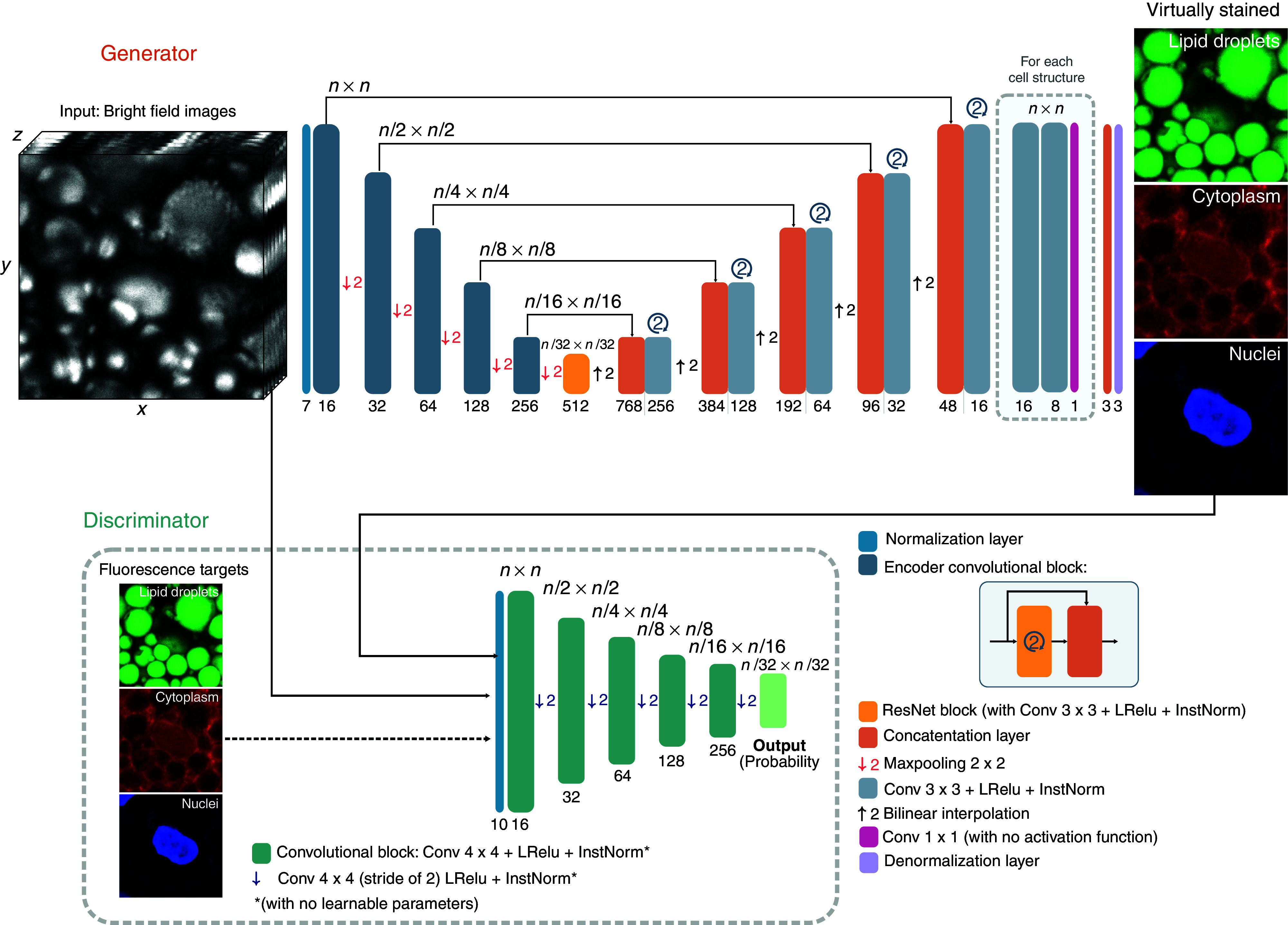
Conditional generative adversarial neural network (cGAN) for virtual staining. The *generator* transforms an input stack of bright-field images into virtually stained fluorescence images of lipid droplets, cytoplasm, and nuclei, using a U-Net architecture with the most condensed layer being replaced by two residual network (ResNet) blocks.^39^ In the first layer of the generator, we normalize each input channel (i.e., each brighfield *z*-slice) in the range [–1,1] using Eq. (B1). The U-Net encoder consists of special convolutional blocks followed by max-pooling layers for downsampling. Each convolutional block contains two paths (a sequence of two ResNet blocks and the identity operation), which are merged by concatenation. The U-Net decoder uses bilinear interpolations for upsampling, followed by concatenations layers and convolutional blocks. Next, one branch is created for each output feature. Each of these branches include two 3 × 3 convolutional blocks, followed by a single 1 × 1 convolutional layer. Each branch is then concatenated back together. In the last layer of the U-Net, the network learns to denormalize the output images back to original pixel values by scaling and adding an offset to the output. Every layer in the generator, except the denormalization layer, the final 1 × 1 convolutional layer, and the pooling layers, is followed by an instance normalization and a leaky ReLU activation. The *discriminator* is designed similar to the PatchGan discriminator^40^ and receives both the bright-field images and fluorescence images (either the target fluorescence images or those predicted by the generator). The inputs to the discriminator are normalized as those to the generator. The discriminator's convolutional blocks consist of 4 × 4 strided convolutions for downsampling. In all layers in the discriminator, we use instance normalization (with no learnable parameters) and leaky ReLU activation. Finally, the discriminator outputs a matrix containing the predicted probability for each patch of 32 × 32 pixels.

## Data Availability

The validation data and software that support the findings of this study are openly available on our Github repository.^25^

## APPENDIX A: ADIPOCYTE CELL CULTURE, IMAGING, AND CELL PROFILING

Adipocytes, or fat cells, are the primary cell type composing adipose tissue. They store energy in the form of lipids, mainly triglycerides, in organelles called lipid droplets. Adipocyte cell cultures are commonly employed to study how the adipocyte metabolic profile responds to therapies for metabolic diseases such as diabetes and nonalcoholic fatty liver disease.^34^ They are also important therapeutically as they are present in the subcutaneous skin layers, and many relatively complex therapeutics, such as nanomedicines, vaccines, or biologicals, are dosed using subcutaneous injections. For example, in the case of nanomedicines and vaccines containing mRNA, the adipocytes are important for creating the active therapeutic protein product.^35^

Human adipose sampling, stem-cell isolation, and subsequent cellular differentiation are described in detail elsewhere.^36^ Briefly, to remove mature adipocytes and isolate stem cells, adipose biopsies are minced, digested, filtered, and centrifuged. For differentiation into adipocytes, 90% confluent stem-cell cultures are treated with DMEM/F12 containing 3% fetal calf serum (Gold; PAA) and supplemented with 100 nM dexamethasone (Sigma), 500 *μ*M 3-isobutyl-1-methyxanthine (Sigma), 0.85 *μ*M insulin, and 5 nM triiodothyronine (Sigma). Media are changed every other day during proliferation and differentiation, until the cells are fully differentiated (day 32).

The mature adipocyte cultures, fixed using 4% paraformaldehyde, are chemically stained to label lipid droplets (Bodipy, green fluorescent), cell cytoplasm (Cell Tracker Deep Red, red fluorescent), and nuclei (Hoechst 33342, blue fluorescent). All fluorescent reagents are from Thermo Fisher Scientific and are used according to the manufacturer's instructions.

The cell cultures are imaged using a robotic confocal microscope (Yokogawa CV7000) equipped with a 60× water-immersion objective (Olympus, UPLSAPO 60XW, NA = 1.2) for the 60× magnification and a 16-bit camera (Andor Zyla 5.5). Illumination correction is applied during acquisition so that the fluorescence intensities are consistent over the field of view. In each well, bright-field and fluorescence images are captured for 12 non-overlapping fields of view (280 μm × 230 μm, 2560 × 2160 pixels), for a total of 96 fields of view for the 60× magnification. For each field of view, a set of four images (one bright-field image and three fluorescence images for lipid droplets, cytoplasm, and nuclei) is acquired at 7 different *z*-positions separated by 1 *μ*m. Subsequently, the fluorescence images at different *z*-positions are projected onto a single image using a maximum intensity projection to create a single fluorescence image per field of view and fluorescence channel. For 40× and 20× magnification, bright-field–fluorescence pairs are acquired for 64 (420 *μ*m × 350 *μ*m) and 48 (830 *μ*m × 700 *μ*m) fields of view using 40× and 20× air objectives (Olympus, UPLSAPO 40× NA = 0.95, UPLSAPO 20× NA = 0.75), respectively.

Using the maximum intensity projections of the confocal fluorescence images, semi-quantitative phenotypic data are extracted from cell structures using the open-source cytometric image analysis software CellProfiler (https://cellprofiler.org, version 4.07^30^) and a custom-made analysis pipeline (the analysis pipelines are available on our Github repository^25^) Measured parameters include object numbers (nuclei, cells, lipid droplets), morphological characteristics (areas), and intensity data.

## APPENDIX B: NEURAL NETWORK ARCHITECTURE

Neural networks are one of the most successful tools for machine learning.^8,37^ They consist of a series of layers of interconnected artificial neurons. These artificial neurons are simple computational units that, when appropriately trained, output increasingly meaningful representations of the input data leading to the sought-after result. Depending on the problem, the architecture of the neural network varies. In particular, generative adversarial networks (GANs)^38^ have been shown to perform well in image-to-image transformation tasks, including recently to realize virtual stainings.^17,18,20,21,24^ A GAN consists of two networks:^38^ a *generator*, which generates images, and a *discriminator*, which discriminates whether images are real or created by the generator. The *adversarial* aspect refers to the fact that these two networks compete against each other: during the training, the generator progressively becomes better at generating synthetic images that can fool the discriminator, while the discriminator becomes better at discriminating real images from synthetic images.

In this work, we employ a conditional GAN (cGAN).^26^ This flavor of GAN for directed image-to-image transformation is chosen over some more recent alternatives, such as the CycleGAN^41^ or StarGAN,^42,43^ because their specific advantages (primarily multi-domain transformations using a single generator and training on unpaired images) are not directly relevant for this project. A schematic of its architecture is shown in Fig. 5. The generator receives as input a stack of bright-field images of the same field of view acquired at different *z*-positions and generates virtually stained fluorescence images of lipid droplets, cytoplasm, and nuclei. The discriminator attempts to distinguish the generated images from fluorescently-stained samples, classifying them as either real or synthetic data. The *conditional* aspect of the cGAN refers to the fact that the discriminator receives both the bright-field stack and the stained images as inputs. Thus, the task of the discriminator is *conditioned* on the bright-field images, i.e., instead of answering “is this a real staining?”, the discriminator answers “is this a real staining *for this stack of bright-field images*?”

In our implementation, the generator is based on the U-Net-architecture,^44^ where the input image is first downsampled to a smaller representation and then upsampled to its original size, with skip connections between the downsampling and upsampling paths to retain local information. We have modified the original U-Net architecture to optimize its performance for virtual staining. First, each encoder convolutional block (Fig. 2) concatenates its input with the result of two sequential ResNet blocks before downsampling; this helps the network to propagate information deeper, because it preserves the input information without the need for the convolutional layers to learn to preserve it. The use of ResNet blocks, where the input of the block is added to the output, is motivated by the need to tackle the vanishing gradient problem and to improve the latent space representation.^14,21,39,45^ This has many of the same advantages as the concatenation step in the encoder blocks, but we have found it to speed up training. Third, every layer (except the final convolutional layer, as well as the denormalization layer and the pooling layers) use instance normalization and a leaky ReLU activation (defined as Φ(x)=α·x, where *α* = 1 for *x *>* *0 and *α* = 0.1 for *x *<* *0), which, differently from standard ReLU, has the advantage of retaining a gradient in the backpropagation step even for negative layer outputs.^46^ Fourth, to help de-correlate the features before the final output of the generator, the final three convolutional layers are evaluated for each output feature independently.

In the first layer of the generator, we normalize the input bright-field *z*-stack as

$${\hat{x}}^{i}=\text{tanh}\left[2\frac{x^{i}-q_{1}^{i}}{q_{99}^{i}-q_{1}^{i}}-1\right],$$
(B1)

where *x^i^* is the pixel value of the *i*th *z*-slice of the original stack and ${\hat{x}}^{i}$ is that of the rescaled *z*-slice, while $q_{p}^{i}$ denotes the *p*th percentile pixel value of that *z*-slice calculated on the entire training set. By estimating the percentiles on the entire training set instead of on a patch-by-patch basis, the normalization becomes more resilient to outliers. Furthermore, by using statistical properties of the distribution of intensities rather than the minimum and maximum of the intensities for normalization, we prevent the normalization from depending on the image size, and we preserve a local correspondence between the intensities of the different channels, which aids the training procedure. Finally, the choice of the hyperbolic tangent as a normalization function ensures that all values fall in the range [−1,1], while mitigating the effect of outliers in the intensity distribution. In the last layer of the U-Net, the network learns to denormalize the output images back to original pixel values by scaling and adding an offset to the output.

We employ a discriminator that follows a conditional PatchGan architecture:^40^ It receives the stack of bright-field images and the fluorescence images (either the target fluorescence images or the virtually stained images), divides them into overlapping patches, and classifies each patch as real or fake (rather than using a single descriptor for the whole input). This splitting arises naturally as a consequence of the discriminator's convolutional architecture.^47^ As shown in Fig. 5, the discriminator's convolutional blocks consist of 4 × 4 convolutional layers followed by strided convolutions for downsampling. In all layers, we use instance normalization (with no learnable parameters) and leaky ReLU activation. Finally, the discriminator output is a matrix that represents the predicted classification probability for each patch. The benefit of using a PathGAN is that the discriminator evaluates the input images based on their style rather than their content. This modification makes the generator task of fooling the discriminator more specialized, thus improving the quality of the generated virtual stainings.^21^

We have implemented this neural network using DeepTrack 2.0, an open-source software for quantitative microscopy using deep learning that we have recently developed,^14,48^ which uses a Python-based TensorFlow backend.^49,50^

## APPENDIX C: TRAINING PROCEDURE

Once the network architecture is defined, we need to train it using *z*-stacks of bright-field images for which we know the corresponding fluorescence target images. As we have seen in Appendix A, the dataset consists of 96 sets of images (each consisting of seven bright-field images and three fluorescence targets with 2560 × 2160 pixels). We randomly split these data into a training dataset and a validation dataset, corresponding to 81 and 15 sets of images, respectively.

Before starting the training process, the bright-field images and corresponding fluorescence targets need to be carefully aligned (a slight misalignment results from the different optics employed to capture the bright-field and fluorescence images). We use a Fourier-space correlation method that calculates a correction factor in terms of a pixel offset and a scale factor (see Appendix D for further details; the code is available on our Github repository^25^) Afterward, we stochastically extract 512 × 512 pixel patches from the corrected images and augment the training dataset using rotational and mirroring augmentations. Importantly, the misalignment must be corrected before the augmentation step because otherwise the augmentations would introduce irreducible errors and put a fundamental limit on high-frequency information.

During training, the trainable parameters of the neural network (i.e., the weights and bias of the artificial neurons in the neural network layers) are iteratively optimized using the backpropagation training algorithm^51^ to minimize the loss function, i.e., the difference between virtually stained images and target chemically stained images. Initially, we set the weights of the convolutional layers of both the generator and discriminator to be randomly (normally) distributed with a mean of 0 and a standard deviation of 0.02; all of the biases are set to 0.

In each training step, we alternately train the generator and the discriminator. First, the generator is tasked with predicting the fluorescence images corresponding to stacks of bright-field images. Then, the discriminator receives both the bright-field images and fluorescence images (either the target fluorescence images or the virtually stained images predicted by the generator) and classifies them as real (chemically stained images, labeled with 1's) or fake (virtually stained images, labeled with 0's).

The loss function of the generator is

$$\begin{array}{c} L_{\text{gen}}=\beta \cdot \text{MAE}\left\{z_{\text{target}},z_{\text{output}}\right\}+\left(1-\beta \right)\cdot {\left(1-D(z_{\text{output}})\right)}^{2}, \end{array}$$
(C1)

where $z_{\text{target}}$ represents the chemically stained (target) images, $z_{\text{output}}$ represents the virtually stained (generated) images, $\text{MAE}\left\{z_{\text{target}},z_{\text{output}}\right\}$ is the mean absolute error between the target and generated images, D(·) denotes is the discriminator prediction, and *β* is a weighting factor between the two part of the loss function (we set β=0.001, which makes the typical value of the MAE roughly half the discriminator term). Importantly, $L_{\text{gen}}$ depends on the discriminator prediction and penalizes the generator for producing images classified as fake. The loss function of the discriminator is

$$L_{\text{disc}}=D{\left(z_{\text{output}}\right)}^{2}+{\left(1-D(z_{\text{target}})\right)}^{2},$$
(C2)

which penalizes the discriminator for misclassifying real images as generated or generated images as real. Thus, the generator tries to minimize its loss by achieving $D(z_{\text{output}})=1$ for the images it generates, while the discriminator tries to achieve $D(z_{\text{output}})=0$ for generated images and $D(z_{\text{target}})=1$ for the chemically stained fluorescence targets. This leads to an adversarial behavior between the generator and the discriminator.

We have trained both networks for 8000 epochs (each of which consisting of 24 batches of 8 images) using the Adam optimizer^52^ with a learning rate of 0.0002 and $\beta_{1}=0.5$ (the exponential decay rate for the 1st moment estimates). Each epoch takes 10 s on a NVIDIA A100 GPU (40 GB VRAM, 2430 MHz effective core clock, 6912 CUDA cores), for a total training time of about 22 h.

## APPENDIX D: IMAGE ALIGNMENT

The bright-field images and the fluorescence images in the original dataset are slightly misaligned. While such misalignment likely does not influence the CellProfiler results on the target images, as the CellProfiler pipeline provides only global information about the images, it strongly influences the local quality of the virtual staining. To quantify this misalignment, we compute the cross correlation of the bright-field images with the lipid droplet images via the Wiener-Khinchin theorem (code can be found on our Github repository^25^). Specifically, the images are divided into patches of 512 × 512 pixels, and their correlation is computed as the inverse Fourier transform of the product of their Fourier transforms

$$C(x,y)=F^{-1}(F(I_{\text{BF}})\bar{F(I_{L})}),$$
(D1)

where $I_{L}$ denotes the lipid image, $I_{\text{BF}}$ denotes the bright-field image, and the bar denotes complex conjugation. An example of this correlation for 20× magnification is shown in Fig. S7. A Gaussian peak is fitted to the resulting correlation map to obtain the misalignment of the two channels, and the offset of this peak relative to (0, 0) defines a misalignment vector $\left(\delta_{x},\delta_{y}\right)$ (arrow in Fig. S7).

Furthermore, by dividing the images into 512 × 512 patches prior to computing the cross correlation allows us to spatially resolve the misalignment within the images. We noticed that there is a constant gradient in the misalignment within each image, consistent with a slight difference in magnification between the bright-field images and the fluorescence images. This is particularly prominent for the samples imaged at 20× magnification, for which the difference in magnification is estimated to be about 0.12%. This is corrected by rescaling the images by a constant factor.

We, therefore, define an affine transformation that has to be applied to the bright-field images in order to align them with the corresponding fluorescence images. This transformation corrects both the misalignment and the difference in magnification. We determine the affine transformation iteratively, by

1. Estimating the affine transformation using the procedure previously explained.
2. Applying the transformation to the bright-field images.
3. Determining the affine transformation again on the transformed images until the affine transformation between the transformed bright-field images and the fluorescence images is close to unitary.

Based on the determined affine transformation, the bright-field images are corrected before training and evaluation.

## APPENDIX E: IMAGE QUALITY METRICS

To quantify the deviation between virtually stained and chemically stained images, we calculated the normalized mean absolute error (nMAE_px_), the structural similarity index measure (SSIM), and the peak signal-to-noise ratio (PSNR) between the two image sets.

The nMAE_px_ is calculated for each image by normalizing the image MAE by the average target value. It is helpful to ensure the network is reproducing the intensity distribution of the images accurately.

The SSIM provides insights into the performance of the network in terms of structural information and is calculated as

$$\text{SSIM}(x,y)=\frac{\left(2\mu_{x}\mu_{y}+c_{1})(2\sigma_{xy}+c_{2}\right)}{\left(\mu_{x}^{2}\mu_{y}^{2}+c1)(\sigma_{x}^{2}\sigma_{y}^{2}+c_{2}\right)},$$
(E1)

where *x* and *y* are the virtually stained and chemically stained image pairs, *μ_x_* and *μ_y_* (*σ_x_* and *σ_y_*) are the mean (standard deviation) of each of the image, *σ_xy_* is the covariance of the images, and *c*_1_ and *c*_2_ are constants to avoid division by a small denominator. The SSIM value lies in the interval [−1,1], being 1 a perfect similarity.

Finally, the PSNR is defined from the root mean square error (RMSE) and is calculated as

$$\text{PSNR}=20\cdot \;{\text{log}}_{10}\left(\frac{L-1}{\text{RMSE}}\right),$$
(E2)

where *L* is the maximum possible intensity level of the image. This metric compares the signal and noise characteristics between virtually stained and chemically stained images.

The nMAE_px_, SSIM, and PSNR values for individual images were averaged to find the average values over the whole validation dataset shown in Table I.
